# Analysis of glycero-lysophospholipids in gastric cancerous ascites[S]

**DOI:** 10.1194/jlr.P072090

**Published:** 2017-03-29

**Authors:** Shigenobu Emoto, Makoto Kurano, Kuniyuki Kano, Keisuke Matsusaki, Hiroharu Yamashita, Masako Nishikawa, Koji Igarashi, Hitoshi Ikeda, Junken Aoki, Joji Kitayama, Yutaka Yatomi

**Affiliations:** Department of Surgical Oncology,* University of Tokyo, Tokyo, Japan; Departments of Clinical Laboratory Medicine† and Gastrointestinal Surgery, University of Tokyo, Tokyo, Japan; # Graduate School of Medicine, University of Tokyo, Tokyo, Japan; CREST,‡ Japan Science and Technology Corporation (JST); Laboratory of Molecular and Cellular Biochemistry,§ Graduate School of Pharmaceutical Sciences, Tohoku University, Miyagi, Japan; Kanamecho Hospital,‖ Tokyo, Japan; Bioscience Division,** TOSOH Corporation, Kanagawa, Japan; Department of Gastrointestinal Surgery,†† Jichi Medical University, Tochigi, Japan

**Keywords:** ascites, gastric cancer, cirrhosis, autotaxin

## Abstract

Lysophosphatidic acid (LysoPA) has been proposed to be involved in the pathogenesis of various cancers. Moreover, glycero-lysophospholipids (glycero-LysoPLs) other than LysoPA are now emerging as novel lipid mediators. Therefore, we aimed to elucidate the possible involvement of glycero-LysoPLs in the pathogenesis of gastric cancer by measuring glycero-LysoPLs, autotaxin (ATX), and phosphatidylserine-specific phospholipase A1 (PS-PLA_1_) in ascites obtained from patients with gastric cancer and those with cirrhosis (as a control). We observed that after adjustments according to the albumin levels, the lysophosphatidylserine (LysoPS) and lysophosphatidylglycerol (LysoPG) levels were significantly higher, while the LysoPA and ATX levels were lower, in the ascites from patients with gastric cancer. We also found that multiple regression analyses revealed that ATX was selected as a significant explanatory factor for all the detectable LysoPA species only in the cirrhosis group and that a significant positive correlation was observed between LysoPS and PS-PLA_1_ only in the gastric cancer group. In conclusion, the LysoPA levels might be determined largely by LysoPC and LysoPI (possible precursors) and the PS-PLA_1_-mediated pathway might be involved in the production of LysoPS in gastric cancer. Glycero-LysoPLs other than LysoPA might also be involved in the pathogenesis of cancer directly or through being converted into LysoPA.

Glycero-lysophospholipids (glycero-LysoPLs) possess a glycerol backbone with one fatty-acid chain and one hydrophilic compartment. This molecular family includes lysophosphatidic acid (LysoPA), lysophosphatidylcholine (LysoPC), lysophosphatidylethanolamine (LysoPE), lysophosphatidylglycerol (LysoPG), lysophosphatidylinositol (LysoPI), and lysophosphatidylserine (LysoPS) (1, 2). Among these glycero-LysoPLs, LysoPA has been most studied in various fields, including oncology; LysoPA works on six kinds of G protein**-**coupled receptors (GPCRs), LysoPA receptors 1–6, located on the cell membrane and reportedly stimulates proliferation, migration, invasion, tumor angiogenesis, and resistance to the chemotherapy of cancer cells (3–6). A series of clinical studies also reported that LysoPA receptors (7–9) and LysoPA-producing enzyme [autotaxin (ATX)] (10–13) are produced or expressed to a greater degree in cancers (5).

In addition to LysoPA, several studies have reported that other glycero-LysoPLs might also be involved in the pathogenesis of cancers. One possible role of glycero-LysoPLs other than LysoPA might be that of precursors to LysoPA, which is known to promote cancer progression. Although LysoPC is the main substrate for ATX in circulation, other glycero-LysoPLs can be hydrolyzed into LysoPA (14). Several specific GPCRs against glycero-LysoPLs other than LysoPA have recently been identified (15), and the direct effects of these glycero-LysoPLs, in addition to their roles as precursors of LysoPA, have been emerging, especially from basic research. For example, LysoPI is reportedly involved in cell proliferation, migration, and survival (16); LysoPE causes migration and the invasion of ovarian cancer cells (17); and LysoPS stimulates the migration of colorectal cancer cells and glioma cells (18, 19). Because specific receptors or producing enzymes could be useful targets for new medicines, glycero-LysoPLs, including LysoPA, are attracting attention in the field of oncology.

Contrary to the possible association between glycero-LysoPLs and cancers, which has been proposed based mainly on the results of basic research, clinical evidence of the involvement of glycero-LysoPLs, even LysoPA, in cancers remains insufficient at present. One possible reason is that the collection of blood samples suitable for analyses of glycero-LysoPLs is rather difficult, because the presence of large amounts of LysoPA precursors, such as LysoPC, or platelet activation can increase the levels of glycero-LysoPLs, especially LysoPA, during blood sampling (20).

With these backgrounds in mind, the present study attempted to elucidate the possible involvement of glycero-LysoPLs in the pathogenesis of cancer in human subjects. For this purpose, we used LC-MS/MS to measure the levels of glycero-LysoPLs and their producing enzymes in ascites collected from subjects with gastric cancer or cirrhosis (as a control).

## METHODS

### Ascites from patients suffering from gastric cancer or cirrhosis

Ascites samples were collected from subjects with advanced gastric cancer (n = 48) or cirrhosis (n = 23), as a control group, for the purpose of palliative care at Kanamecho Hospital (Tokyo, Japan). Because the existence of ascites in healthy subjects is limited and the procedure for its sampling is quite invasive, we utilized the ascites from subjects with cirrhosis, in which malignant cells were not included, as a control to investigate the characteristics of malignant ascites.

The supernatant components were collected by centrifugation and stored in aliquots at −80°C, then subjected to one freeze-thaw cycle before the measurement of the glycero-LysoPLs, ATX, and phosphatidylserine-specific phospholipase A1 (PS-PLA_1_). The present study was conducted with the approval of the ethics review committee of the University of Tokyo, and all the participants signed informed consent forms.

### Measurement of glycero-LysoPLs, ATX, and PS-PLA_1_ in the ascites

The glycero-LysoPLs were quantified using LC-MS/MS, as previously described (21, 22). Briefly, the plasma samples were mixed and sonicated with methanol and an internal standard (1 μM 17:0 LysoPA or 10 μM 17:0 LysoPC). After centrifugation at 21,500 *g*, the resulting supernatant was recovered and used for the LC-MS analysis. Then, 20 μl of the methanol extract was separated using a Nanospace LC (Shiseido) equipped with a C18 CAPCELL PAK ACR column (1.5 × 250 mm; Shiseido) using a gradient of solvent A (5 mM ammonium formate in water) and solvent B [5 mM ammonium formate in 95% (v/v) acetonitrile]. The eluate was sequentially ionized using an ESI probe, and the parent ion (*m/z* 380.2) and the fragment ion (*m/z* 264.2) were monitored in the positive mode using a Quantum Ultra triple quadrupole mass spectrometer (Thermo Fisher Scientific). For each LysoPL class, 12 acyl chains (14:0, 16:0, 16:1, 18:0, 18:1, 18:2, 18:3, 20:3, 20:4, 20:5, 22:5, and 22:6) were monitored. We calculated the concentrations of LysoPLs from the area ratio to the internal standard: 1 μM 17:0 LysoPA (for LysoPA, LysoPE, LysoPI, LysoPG, and LysoPS species) or 10 μM 17:0 LysoPC (for LysoPC species).

The ATX and PS-PLA_1_ antigen levels in the ascites were determined using a two-site immunoenzymetric assay with the TOSOH AIA system (TOSOH, Tokyo, Japan) (23, 24). Regarding ATX, because five alternative splicing isoforms of ATX have been identified as ATXα, ATXβ, ATXγ, ATXδ, and ATXε, we also measured the classical ATX (ATXα, ATXβ, and ATXγ) and novel ATX (ATXδ and ATXε) levels using enzyme immunoassays that we recently developed (25).

### Statistical analysis

All the data were statistically analyzed using SPSS (Chicago, IL). The results are expressed as the mean ± 2SD. We performed nonparametric analyses, because normality or equality of variance was rejected by the Kolmogorov-Smirnov test or the Levene test for most of the parameters or analyses; a comparison between two groups was performed using the Mann-Whitney U test and correlations were determined using the Spearman correlation test. The independent effects of the glycero-LysoPLs and the total ATX level on LysoPA were evaluated using a stepwise multiple regression analysis. A value of *P* < 0.05 was regarded as denoting statistical significance in all the analyses.

## RESULTS

### Elevated LysoPS and LysoPG levels and lower LysoPA levels in ascites from patients with gastric cancer compared with those in ascites from patients with cirrhosis after adjustments according to the albumin level

**Fig. 1.**
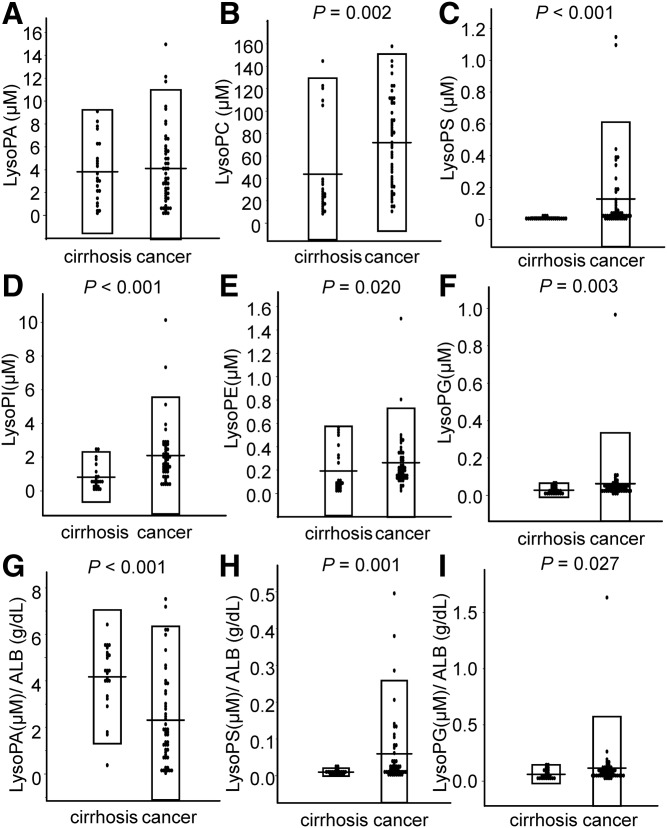
Glycero-LysoPL levels in ascites from patients with cirrhosis or gastric cancer. Using LC-MS/MS, the glycero-LysoPL levels were measured in ascites from patients with cirrhosis (n = 23) and from patients with gastric cancer (n = 48). Total LysoPA levels (A), total LysoPC levels (B), total LysoPS levels (C), total LysoPI levels (D), total LysoPE levels (E), total LysoPG levels (F), total LysoPA levels (G) adjusted according to the ALB level, total LysoPS levels adjusted according to the ALB level (H), and total LysoPG levels adjusted according to the ALB level (I).

First, we measured the concentration of glycero-LysoPLs in ascites collected from subjects with gastric cancer and cirrhosis (**Fig. 1A–F**). The concentrations of total LysoPC were 4.36 ± 8.59 μM in cirrhosis versus 7.18 ± 7.90 μM in cancer (*P* = 0.002); those of total LysoPS were 0.00773 ± 0.0109 μM in cirrhosis versus 0.128 ± 0.484 μM in cancer (*P* < 0.001); those of total LysoPI were 0.817 ± 1.49 μM in cirrhosis versus 2.10 ± 3.46 μM in cancer (*P* < 0.001); those of LysoPE were 0.192 ± 0.383 μM in cirrhosis versus 0.263 ± 0.467 μM in cancer (*P* = 0.020); and those of LysoPG were 0.0277 ± 0.038 μM in cirrhosis versus 0.0626 ± 0.271 μM in cancer (*P* = 0.001). Accordingly, the concentrations of total LysoPC, LysoPS, LysoPI, LysoPE, and LysoPG were significantly higher in the ascites from the subjects with gastric cancer, while the concentration of LysoPA was not different between these groups.

Because the ascites from subjects with cancer are exudates, while the ascites caused by cirrhosis are transudates, we measured the concentrations of total protein (TP) and albumin (ALB). The concentrations of TP were 1.98 ± 2.51 g/dl in cirrhosis versus 3.57 ± 2.71 g/dl in cancer (*P* < 0.001), while those of ALB were 0.88 ± 1.12 g/dl in cirrhosis versus 1.94 ± 1.73 g/dl in cancer (*P* < 0.001), confirming that the concentrations of TP and ALB were higher in the ascites from the subjects with gastric cancer. Therefore, we next adjusted the concentrations of glycero-LysoPLs to ALB to investigate whether the differences simply reflected the general difference in the characteristics of ascites (exudates or transudates) or whether the different pathophysiological factors related to each disease were involved. The ratios of LysoPS/ALB were 0.0097 ± 0.0107 μM/g/dl in cirrhosis versus 0.0591 ± 0.199 μM/g/dl in cancer (*P* = 0.001) and those of LysoPG/ALB were 0.061 ± 0.082 μM/g/dl in cirrhosis versus 0.115 ± 0.459 μM/g/dl in cancer (*P* = 0.027), while the LysoPA level was significantly higher in the ascites caused by cirrhosis [4.17 ± 2.88 μM/g/dl in cirrhosis vs. 2.37 ± 3.97 μM/g/dl in cancer (*P* < 0.001)] (Fig. 1G–I, supplemental Figure S1). Accordingly, when adjusted to ALB, the differences in the LysoPC and LysoPI levels were not observed, while the LysoPS and LysoPG levels remained higher in the ascites from gastric cancer.

**Fig. 2.**
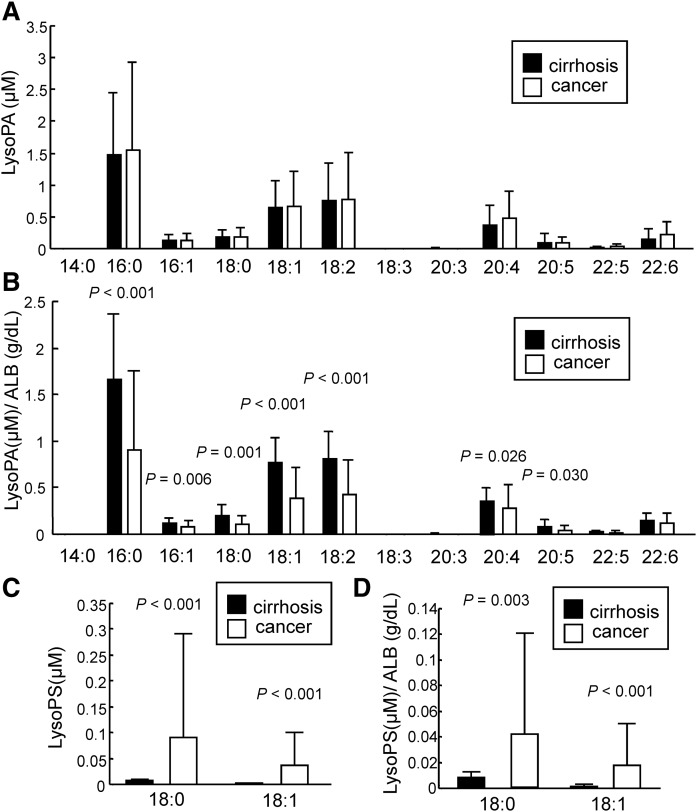
LysoPA species and LysoPS species in ascites from patients with cirrhosis or gastric cancer. The levels of LysoPA species (A, B) and LysoPS species (C, D) were measured as described in Fig. 1. B, D: The LysoPA and LysoPS levels were adjusted according to the ALB level.

Recent basic studies have demonstrated differences in the bioactivities among molecular species of glycero-LysoPLs, so we next compared the molecular species of glycero-LysoPLs in the ascites samples. As shown in **Fig. 2A, B**, the unadjusted levels of all LysoPA species were not different, while the ALB-adjusted levels were higher in the cirrhosis group with the exception of 22:5 and 22:6 LysoPA. Compared with the pattern of LysoPA species in human plasma (22), 16:0 LysoPA was especially dominant in the ascites. The LysoPC and LysoPI levels were higher in the gastric cancer group without adjustments for ALB, while significant differences in most of the LysoPC and LysoPI species were not observed after adjustment according to the ALB level except for the 18:2 and 20:4 LysoPC levels (supplemental Figs. S2A, B, S3A, B). We also observed that the LysoPS and LysoPG levels for all of the molecular species detected in the ascites were higher in the ascites from gastric cancers (Fig. 2C, supplemental Fig. S2C) and that the levels of all of the LysoPS species and several LysoPG species were still higher in the ascites from gastric cancers after adjustment according to the ALB level (Fig. 2D, supplemental Fig. S3C). Of note, only the 18:0 and 18:1 species of LysoPS were detected in the ascites, while many other species were detected in the plasma samples (22). Regarding the LysoPE species, the levels of 16:0, 18:0, and 22:6 LysoPE were higher in the ascites from the gastric cancer group before adjustment, while the levels of 18:0 and 18:2 LysoPE were lower in the gastric cancer group after adjustment according to the ALB level (supplemental Figs. S2D, S3D).

### Significantly higher concentrations of ATX in ascites from patients with cirrhosis than in ascites from patients with gastric cancer

**Fig. 3.**
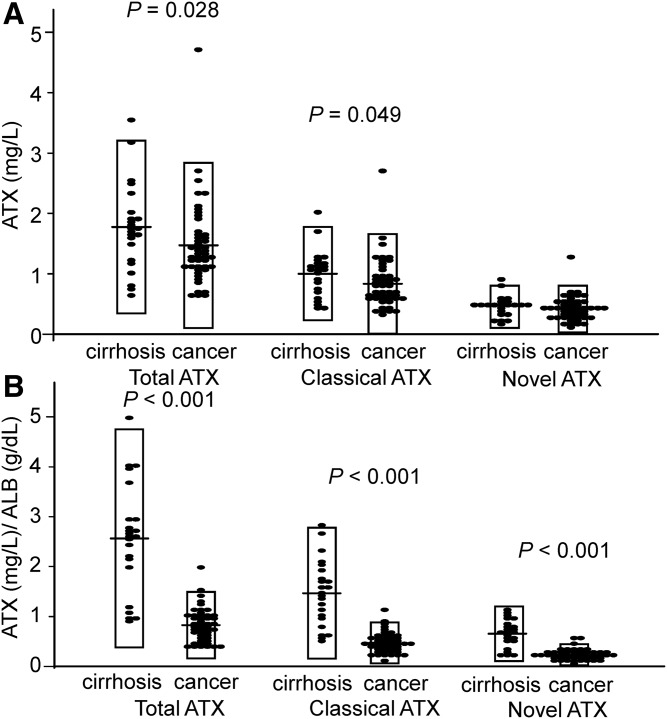
ATX levels in ascites from patients with cirrhosis or gastric cancer. A: The total ATX, classical ATX, and novel ATX levels were measured in the samples as described in Fig. 1. B: The total ATX, classical ATX, and novel ATX levels were adjusted according to the ALB level.

Because we observed different levels of LysoPA in ascites from patients with cirrhosis and in ascites from patients with gastric cancer, we measured the concentrations of ATX (a LysoPA-producing enzyme). As shown in **Fig. 3A, B**, the total ATX and classical ATX levels were marginally, but significantly, higher in ascites from patients with cirrhosis and, when adjusted according to the ALB level, the total ATX, classical ATX, and novel ATX levels were all considerably higher in the ascites from patients with cirrhosis compared with the levels in ascites from patients with gastric cancer.

### LysoPA levels were significantly and positively correlated with the ATX levels in ascites from patients with cirrhosis and in ascites from patients with gastric cancer

**Fig. 4.**
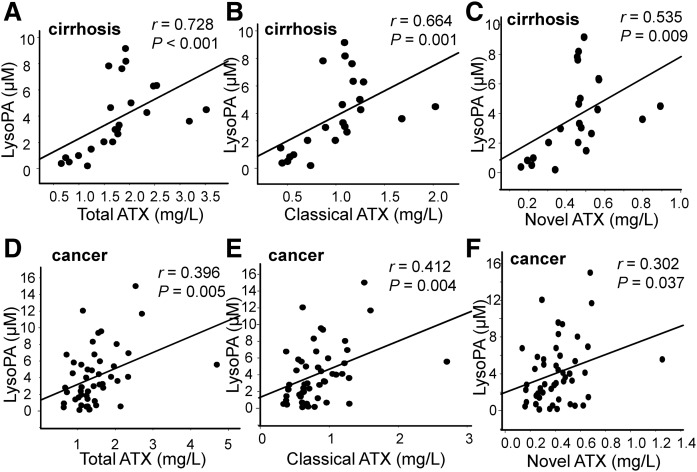
Correlation between the LysoPA and ATX levels in ascites from patients with cirrhosis or gastric cancer. The correlations between the total LysoPA level and the total ATX (A, D), classical ATX (B, E), and novel ATX (C, F) levels in ascites from patients with cirrhosis (A–C) or from patients with gastric cancer (D–F) are shown.

Because the plasma LysoPA levels are strongly correlated with the ATX levels in healthy subjects (26), subjects with chronic liver diseases (27), and subjects with follicular lymphoma (11), we next investigated the correlation between the LysoPA and ATX levels. As shown in **Fig. 4A–F**, significant strong correlations were observed between the total LysoPA levels and the total ATX (*r* = 0.728, *P* < 0.001) and moderate correlations were observed between the total LysoPA and classical ATX or novel ATX levels (*r* = 0.664, *P* < 0.001 and *r* = 0.535, *P* = 0.009, respectively) in ascites from patients with cirrhosis, while only weak to moderate correlations were observed in ascites from patients with cancer. We did not find any specific correlation between the LysoPA molecular species and the total ATX levels, as shown in supplemental Table S1.

### LysoPA levels were significantly correlated with the levels of possible precursor glycero-LysoPLs in ascites from patients with cirrhosis and in ascites from patients with gastric cancer

**Fig. 5.**
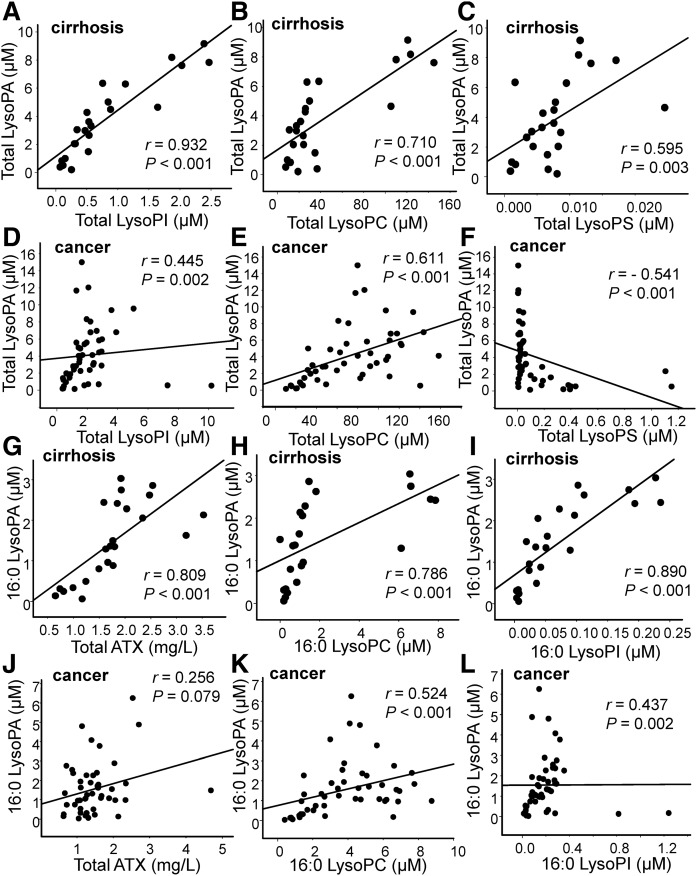
Correlation between the LysoPA level and other glycero-LysoPL levels in ascites from patients with cirrhosis or gastric cancer. The correlations between the total LysoPA level (A–F) or the 16:0 LysoPA level (G–L) and the total LysoPI (A, D), total LysoPC (B, E), total LysoPS (C, F), total ATX (G, J), 16:0 LysoPC (H, K), or 16:0 LysoPI (I, L) level in ascites from patients with cirrhosis (A–C, G–I) or from patients with gastric cancer (D–F, J–L) are shown.

When we previously investigated the sources of elevated plasma LysoPA levels in subjects with acute coronary syndrome, the levels of LysoPA precursors appeared to determine the plasma LysoPA levels (22). Therefore, we next investigated the correlations between LysoPA and its possible precursors, LysoPC, LysoPI, LysoPE, LysoPG, and LysoPS. As shown in **Fig. 5A–C** and supplemental Fig. S4A, B, the total LysoPA level was strongly correlated with the levels of LysoPI and LysoPC (*r* = 0.932, *P* < 0.001 and *r* = 0.710, *P* < 0.001, respectively), moderately correlated with those of LysoPS and LysoPE (*r* = 0.595, *P* = 0.003 and *r* = 0.690, *P* < 0.001, respectively), and uncorrelated with those of LysoPG (*r* = 0.396, *P* = 0.061) in ascites from patients with cirrhosis, while the total LysoPA level was moderately correlated with the LysoPC, LysoPI, and LysoPE levels (*r* = 0.445, *P* = 0.002; *r* = 0.611, *P* < 0.001; and *r* = 0.488, *P* < 0.001; respectively) and, interestingly, negatively correlated with the LysoPS level in ascites from patients with gastric cancer (Fig. 5D–F; supplemental Fig. S4C, D). Regarding the correlations between the LysoPA species and the corresponding glycero-LysoPL species, we observed similar correlations (Fig. 5 G–L; supplemental Tables S2, S3), suggesting that glycero-LysoPLs, especially LysoPC, LysoPI, and LysoPE, might serve as precursors for LysoPA in the ascites.

### Possible difference in determinants for LysoPA between ascites from patients with cirrhosis and ascites from patients with gastric cancer

Although the correlations between LysoPA and ATX or its possible precursor glycero-LysoPLs showed almost the same tendency, except for LysoPS, the strength of the correlations between the LysoPA and ATX levels seemed to differ between the two groups. Therefore, we next performed multiple regression analyses using the LysoPA level as the objective variable and the levels of total ATX and glycero-LysoPLs other than LysoPA as possible explanatory variables. As shown in **Tables 1**, **2**, ATX was selected as a significant explanatory factor for all the LysoPA species in the cirrhosis group, but only for 18:2 LysoPA in the gastric cancer group. The glycero-LysoPLs selected as significant explanatory factors also differed somewhat: the LysoPI species was selected for 16:0, 18:1, and 20:4 LysoPA in the cirrhosis group, while it was selected only for 20:4 LysoPA in the gastric cancer group. In contrast, the LysoPC species was selected as a significant explanatory factor for 16:0, 18:2, 20:4, and 22:6 LysoPA in the gastric cancer group, but only for 18:2 and 22:6 LysoPA in the cirrhosis group.

**TABLE 1.** Multiple regression analyses for plasma LysoPA species in ascites from patients with gastric cancer

	B	95% CI	Standardized β	*P*
Total LysoPA				
Total LysoPC	0.039	0.019–0.060	0.454	<0.001
Total LysoPS	−5.270	−8.679 to −1.861	−0.371	0.003
16:0 LysoPA				
16:0 LysoPC	0.021	0.004–0.038	0.338	0.019
18:1 LysoPA				
18:1 LysoPS	−3.982	−6.187 to −1.778	−0.456	0.001
ATX	0.212	0.007–0.417	0.261	0.043
18:2 LysoPA				
18:2 LysoPC	0.246	0.173–0.319	0.707	<0.001
20:4 LysoPA				
20:4 LysoPI	1.950	1.175–2.725	0.592	<0.001
20:4 LysoPC	0.298	0.122–0.475	0.398	0.001
22:6 LysoPA				
22:6 LysoPC	0.271	0.174–0.367	0.642	<0.001

Multiple regression analyses for total LysoPA and LysoPA species in ascites from patients with gastric cancer. The glycero-LysoPLs of the corresponding molecular species and ATX were utilized as possible explanatory factors. B represents the unstandardized coefficients.

**TABLE 2.** Multiple regression analyses for plasma LysoPA species in ascites from patients with cirrhosis

	B	95% CI	Standardized β	*P*
Total LysoPA				
Total LysoPI	3.765	3.082–4.448	1.035	<0.001
ATX	1.068	0.593–1.544	0.283	<0.001
Total LysoPS	−138.2	−229.1 to −47.43	−0.278	0.005
16:0 LysoPA				
16:0 LysoPI	14.996	8.313–21.680	1.131	<0.001
ATX	0.440	0.142–0.738	0.325	0.006
16:0 LysoPE	−8.901	−17.744 to −0.0571	−0.475	0.049
18:1 LysoPA				
18:1 LysoPI	1.925	1.425–2.425	0.772	<0.001
ATX	0.224	0.108–0.340	0.388	0.001
18:2 LysoPA				
18:2 LysoPC	0.294	0.248–0.341	0.889	<0.001
ATX	0.184	0.066–0.303	0.220	0.004
20:4 LysoPA				
20:4 LysoPI	2.235	1.765–2.705	0.835	<0.001
ATX	0.168	0.087–0.248	0.381	<0.001
20:4 LysoPC	−0.083	−0.161 to −0.006	−0.195	0.037
22:6 LysoPA				
22:6 LysoPC	0.274	0.232–0.316	0.913	<0.001
ATX	0.036	0.006–0.067	0.168	0.022

Multiple regression analyses for the total LysoPA and LysoPA species in ascites from patients with cirrhosis. The glycero-LysoPLs of the corresponding molecular species and ATX were utilized as possible explanatory factors. B represents the unstandardized coefficients.

### LysoPS levels were significantly and positively correlated with the PS-PLA_1_ levels only in ascites from patients with gastric cancer

**Fig. 6.**
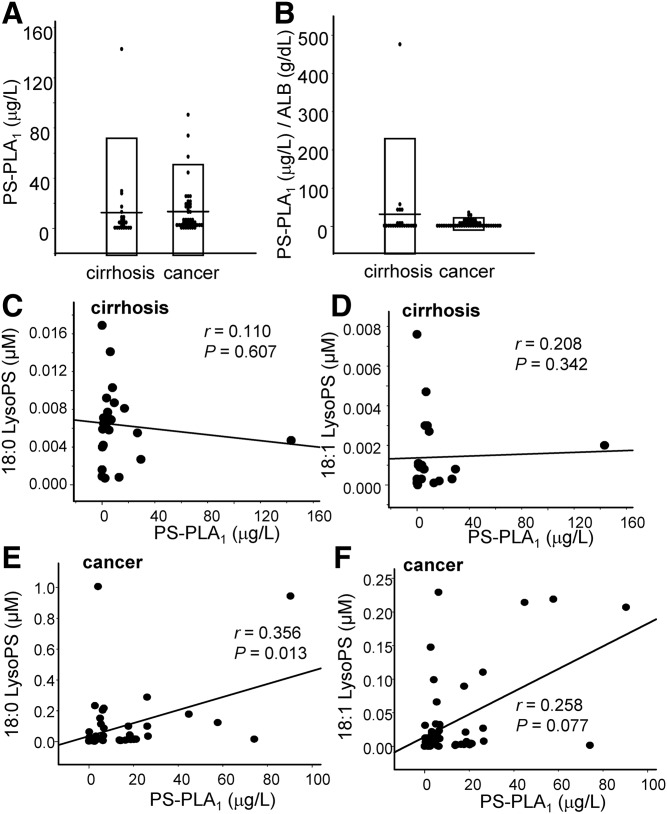
PS-PLA_1_ level and its correlation with the LysoPS level in ascites from patients with cirrhosis or gastric cancer. The PS-PLA_1_ level was measured in the samples as described in Fig. 1 and the correlation between the LysoPS and PS-PLA_1_ levels was investigated. The PS-PLA_1_ levels (A), the PS-PLA_1_ levels adjusted according to the ALB level (B), and the correlations between the 18:0 or 18:1 LysoPS and PS-PLA_1_ levels (C–F) in patients with cirrhosis (C, D) or gastric cancer (E, F) are shown.

In addition to the possible difference in the determinants for LysoPA, we have also observed that the levels of LysoPS, which is an emerging glycero-LysoPL mediator (28), were higher in ascites from patients with gastric cancer (Fig. 1C, H). Therefore, we next measured the PS-PLA_1_ levels, which are thought to produce LysoPS from phosphatidylserine (PS) (2, 29). Contrary to the higher levels of LysoPS in ascites from gastric cancer group, we did not observe any difference in the PS-PLA_1_ levels between the two groups either with or without adjustments to the ALB (**Fig. 6A, B**).

When we investigated the correlation between LysoPS and PS-PLA_1_, however, we found that the total LysoPS and 18:0 LysoPS levels were weakly, but significantly and positively, correlated with the PS-PLA_1_ level only in the ascites from the subjects with gastric cancer (*r* = 0.331, *P* = 0.022 and *r* = 0.356, *P* = 0.013, respectively) (Fig. 6C–F, supplemental Fig. S5), suggesting that the homeostasis of LysoPS might differ between patients with gastric cancer and those with cirrhosis.

## DISCUSSION

Glycero-LysoPLs, represented by LysoPA, are reportedly involved in the pathogenesis of neoplasms: LysoPA has been suggested to accelerate tumor proliferation, migration, invasion, and metastasis (3–6). In addition to LysoPA, other glycero-LysoPLs are emerging as potent lipid mediators, based especially on the findings of basic research, and several receptors for some glycero-LysoPLs have recently been identified (15). Considering the physiological properties of these glycero-LysoPLs, they might be involved in the pathogenesis of cancer as well as LysoPA. Clinical research, however, has provided little evidence of the involvement of glycero-LysoPLs in oncology, even for LysoPA. Therefore, in the present study, we measured the levels of glycero-LysoPLs (LysoPA, LysoPC, LysoPI, LysoPE, LysoPG, and LysoPS) together with ATX and PS-PLA_1_ (producing enzymes for LysoPA and LysoPS, respectively) in ascites from subjects with advanced gastric cancers. We also collected ascites from subjects with cirrhosis as a control.

In this study, we found several possible differences in LysoPA determinants between ascites from patients with cirrhosis and ascites from patients with gastric cancer. Because the correlations between the LysoPA species and ATX seemed weaker (Fig. 4) and ATX was not selected as a significant explanatory factor for most of the LysoPA species using multiple regression analyses for the subjects with gastric cancer (Table 1), the LysoPA levels might not be determined by ATX in ascites from patients with gastric cancer, at least to a great degree. Instead, possible substrates for ATX, especially LysoPC and LysoPI, were selected as significant positive explanatory factors, suggesting that these substrates might determine LysoPA production in patients with gastric cancer. Although the physiological roles of this possible homeostasis of LysoPA in gastric cancer have remained unknown, several cancers reportedly exhibit increases in phosphatidylinositol, phosphatidylethanolamine, and phosphatidylcholine (30–33), as well as phospholipase A2 (34–38), which creates LysoPLs from diacylphospholipids. Considering these reports together with the results from the present study, we can hypothesize that the increased levels of diacylphospholipids might be converted to glycero-LysoPLs (other than LysoPA), which are then hydrolyzed into LysoPA via ATX, promoting the spread of cancer. This possible producing pathway for LysoPA could be a pharmacological target for the treatment of cancer.

Besides LysoPA, we were also interested in the LysoPS levels in gastric cancer, because LysoPS is a novel lipid mediator for which receptors have recently been identified (28). As shown in Fig. 1C, H, the LysoPS level was higher in the gastric cancer group, even after adjustments to the ALB level, while the PS-PLA_1_ level, which is a LysoPS-producing enzyme, was not modulated (Fig. 6A, B). Regarding the correlation between LysoPS and PS-PLA_1_, a significant positive correlation was only observed in the gastric cancer group (supplemental Fig. S5, Fig. 6C–F). These correlations might be attributable to the dynamism of phospholipids in the plasma membrane, because PS, a substrate for PS-PLA_1_, is exposed on the cell membrane in apoptotic cancer cells (39, 40). Actually, we have observed a similar positive correlation between LysoPS and PS-PLA_1_ in subjects with acute coronary syndrome (41). Strong blood coagulation, as is the case in patients with acute coronary syndrome, is another event that, like apoptosis, results in the exposure of PS on the plasma membrane (42). Although the physiological roles of LysoPS in the fields of oncology remain to be elucidated, the immune-suppressive properties of LysoPS that have been reported in several fields other than oncology (43–45) suggest that LysoPS might be involved in the immunological escape that is observed in cancers; when several cancer cells undergo apoptosis, PS is flipped outside the cell membrane and is converted into LysoPS by PS-PLA_1_, possibly suppressing immunological attacks to other living cancer cells in tumor tissues. Further studies are needed to investigate the hypotheses suggested by the present findings.

An important issue that remains to be solved is the correlation between 18:0- and 18:1-containing LysoPS and PS-PLA_1_. Basically, saturated and unsaturated fatty acids attach to the *sn*-1 and *sn*-2 positions of glycero-phospholipids, respectively. Accordingly, the deacylation of the *sn*-1 position of PS by PS-PLA_1_ theoretically results in the formation of 1-lyso, 2-acyl LysoPS, i.e., unsaturated LysoPS (rather than the saturated form) (29), which seems to be inconsistent with our present finding that the PS-PLA_1_ level is also correlated with the saturated LysoPS level. The composition of fatty acids, however, varies depending on the tissue or cell type (46), and an analysis of the fatty acid composition of phosphatidylcholine in lipids extracted from the membrane fractions of mouse tissues reveals their diversity. For example, 16:0- and 22:6-containing phosphatidylcholine are predominant in the heart, while 16:0- and 16:0-containing phosphatidylcholine are predominant in the lungs and undergo further alteration under pathological conditions (47). Given that 18:0- and 18:1-containing PS may be the main fatty acids in gastric cancer cells infiltrating the peritoneum, PS-PLA_1_ and/or unidentified novel phospholipase A2, which deacetylates the *sn*-2 position of PS, might be involved in the formation of 18:0 or 18:1 LysoPS. This finding is also consistent with the fact that 16:0 LysoPA was predominant in the ascites, which is not the case with the pattern of LysoPA species in human plasma. Further studies are needed to elucidate the mechanism by which LysoPS is formed in ascites resulting from the invasion of gastric cancer.

Regarding glycero-LysoPLs other than LysoPA and LysoPS, we observed that the LysoPI and LysoPG levels were more than twice as high as those in the gastric cancer group and that the LysoPG level remained higher even after adjustment according to the ALB level. In addition to its possible role as a precursor of LysoPA, LysoPI might possess direct roles in the pathogenesis of cancers, because GPR55 has been identified as a specific GPCR for LysoPI (48, 49) and LysoPG is also reportedly involved in inflammation (50). Therefore, the results from the present study further suggest the need to investigate the pathological roles of LysoPI and LysoPG in the fields of oncology.

The main limitation of this study is that we used ascites collected from patients with cirrhosis as a control, because it is impossible to collect control ascites from healthy subjects. Considering that the LysoPA and ATX levels are known to be elevated in cirrhosis (51), the present results should be carefully interpreted when comparing the concentrations between the two groups; for example, the finding that the LysoPA level after adjustment according to the ALB level was lower in the gastric cancer group does not necessarily mean that the LysoPA might not be involved in the pathogenesis of gastric cancer. Instead, the finding could indicate that the LysoPA and ATX levels were very high in ascites from patients with cirrhosis. Regardless, we can safely conclude that patients with gastric cancer might possess a distinct glycero-LysoPL homeostasis profile, compared with patients with cirrhosis. We also admit that several samples, of which the concentrations of glycero-LysoPLs, ATX, or PSPLA1 were deviated from the range of the mean ± 3SDs, exist in several figures and might somehow affect the appearance of the correlations: one sample for Figs. 4E, F, 5C, E, K, 6C; two samples for Figs. 4D, 5D, J, 6D and supplemental Figs. S4C, D, S5A; three samples for Figs. 5F, L, 6F; and four samples for Fig. 6E and supplemental Fig. S5B. To avoid the possible influences of these deviated points on the statistical analyses, we have adopted the nonparametric analyses, as shown in the Methods section, and confirmed the similar correlations when these deviated samples are excluded (data not shown).

In summary, in ascites from patients with gastric cancer, the LysoPA levels might be determined largely by its precursors, such as LysoPC and LysoPI, and LysoPS might be produced via a PS-PLA_1_-mediated pathway to a greater degree than in ascites from patients with cirrhosis. These results suggest the possible involvement of glycero-LysoPLs in the pathogenesis of gastric cancer.

## Supplementary Material

Supplemental Data
