# Supplementary material for: Analysis of glycero-lysophospholipids in gastric cancerous ascites
Source: J Lipid Res. 2017 Mar 29;58(4):763–71. doi: 10.1194/jlr.P072090 (PMC5392751; doi:10.1194/jlr.P072090)
Supplement: Supplemental Data [file supp_58_4_763__index.html]

Analysis of glycero-lysophospholipids in gastric cancerous ascites — Analysis of glycero-lysophospholipids in gastric cancerous ascites — Supplemental Data 

# Analysis of glycero-lysophospholipids in gastric cancerous ascites

## Supplemental Data

- Supplemental data (.pdf, 475 KB) - Supplemental figures and tables.
