# Supplementary material for: Analysis of glycero-lysophospholipids in gastric cancerous ascites
Source: J Lipid Res. 2017 Mar 29;58(4):763–71. doi: 10.1194/jlr.P072090 (PMC5392751; doi:10.1194/jlr.P072090)
Supplement: Supplemental Data [file 10.1194_P072090_jlr.P072090-1.pdf]

Supplemental Figure S1

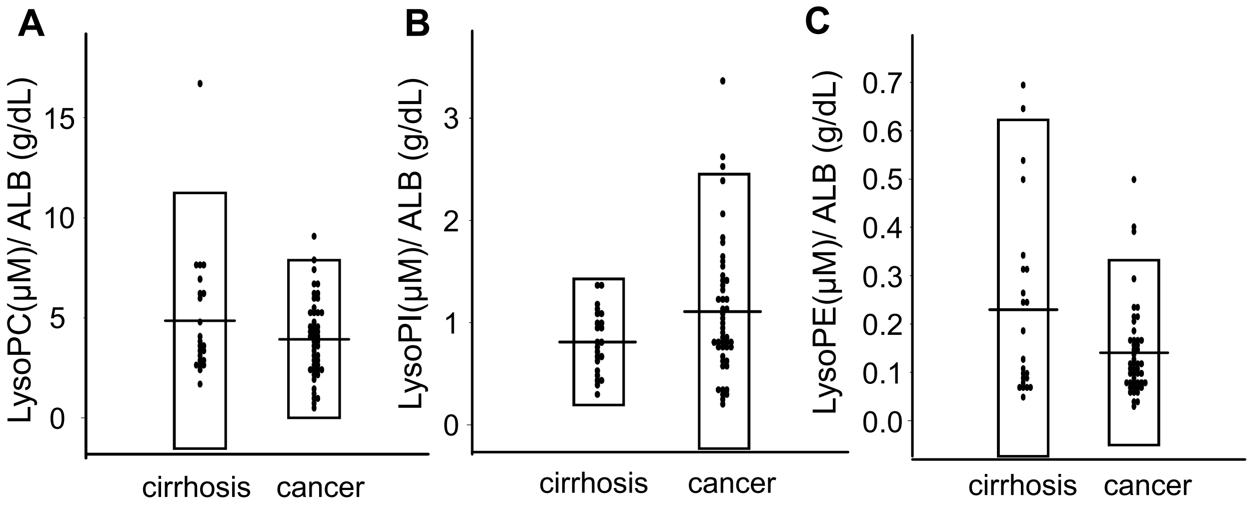

Supplemental Figure S1. Glycero-LPL levels in ascites from patients with cirrhosis or gastric cancer.

The glycero-LPL levels were measured as described in Figure 1. (A) LysoPC levels adjusted according to the albumin level, (B) LysoPI levels adjusted according to the albumin level, and (C) LysoPE levels adjusted according to the albumin level.

Supplemental Figure S2

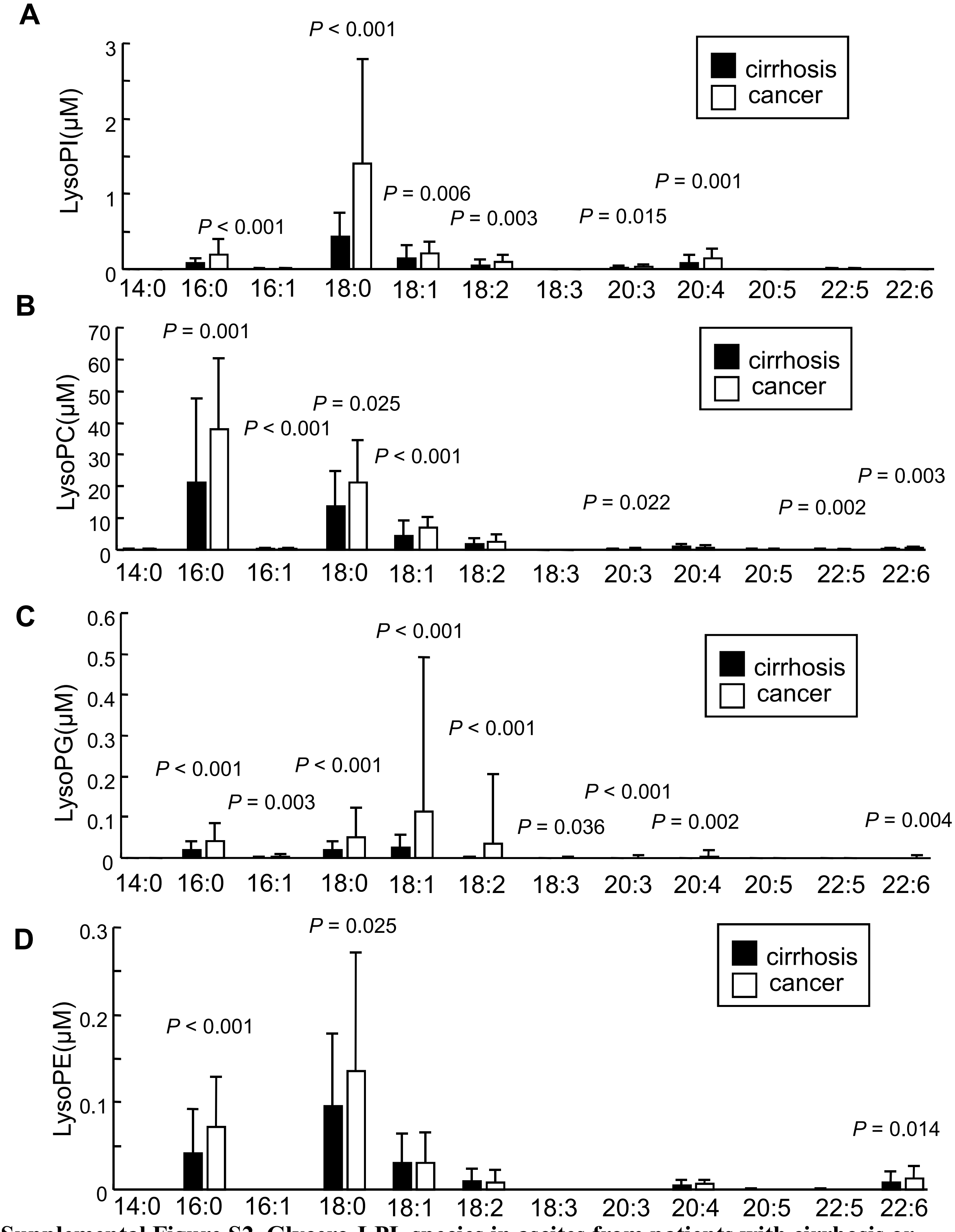

Supplemental Figure S2. Glycero-LPL species in ascites from patients with cirrhosis or gastric cancer.

The levels of LysoPI species (A), LysoPC species (B), LysoPG species (C), and LysoPE species (D) were measured as described in Figure 1.

Supplemental Figure S3

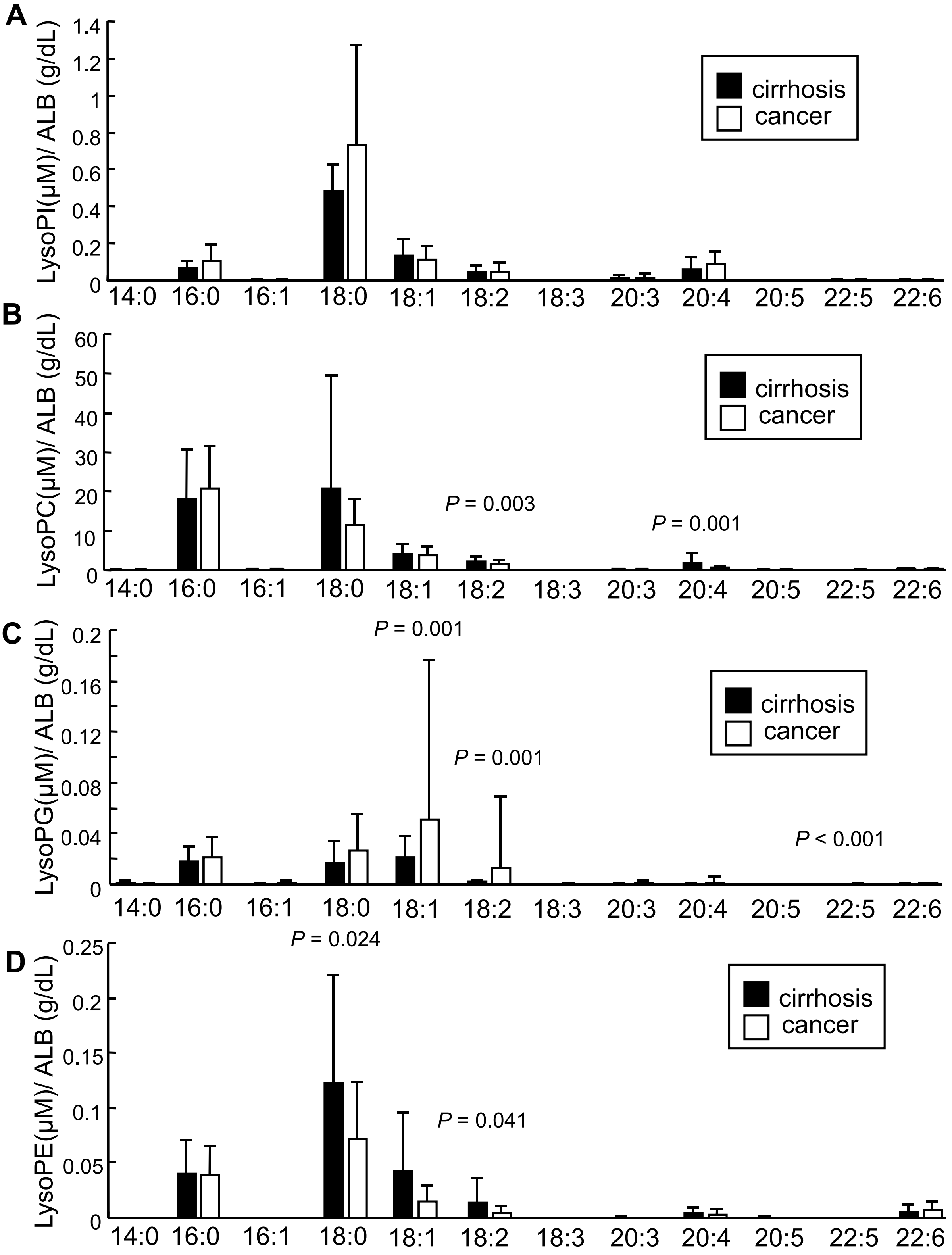

Supplemental Figure S3. Glycero-LPL species adjusted according to the albumin level in ascites from patients with cirrhosis or gastric cancer.

The levels of LysoPI species (A), LysoPC species (B), LysoPG species (C), and LysoPE species (D) were measured as described in Figure 1 and were adjusted according to the albumin level.

Supplemental Figure S4

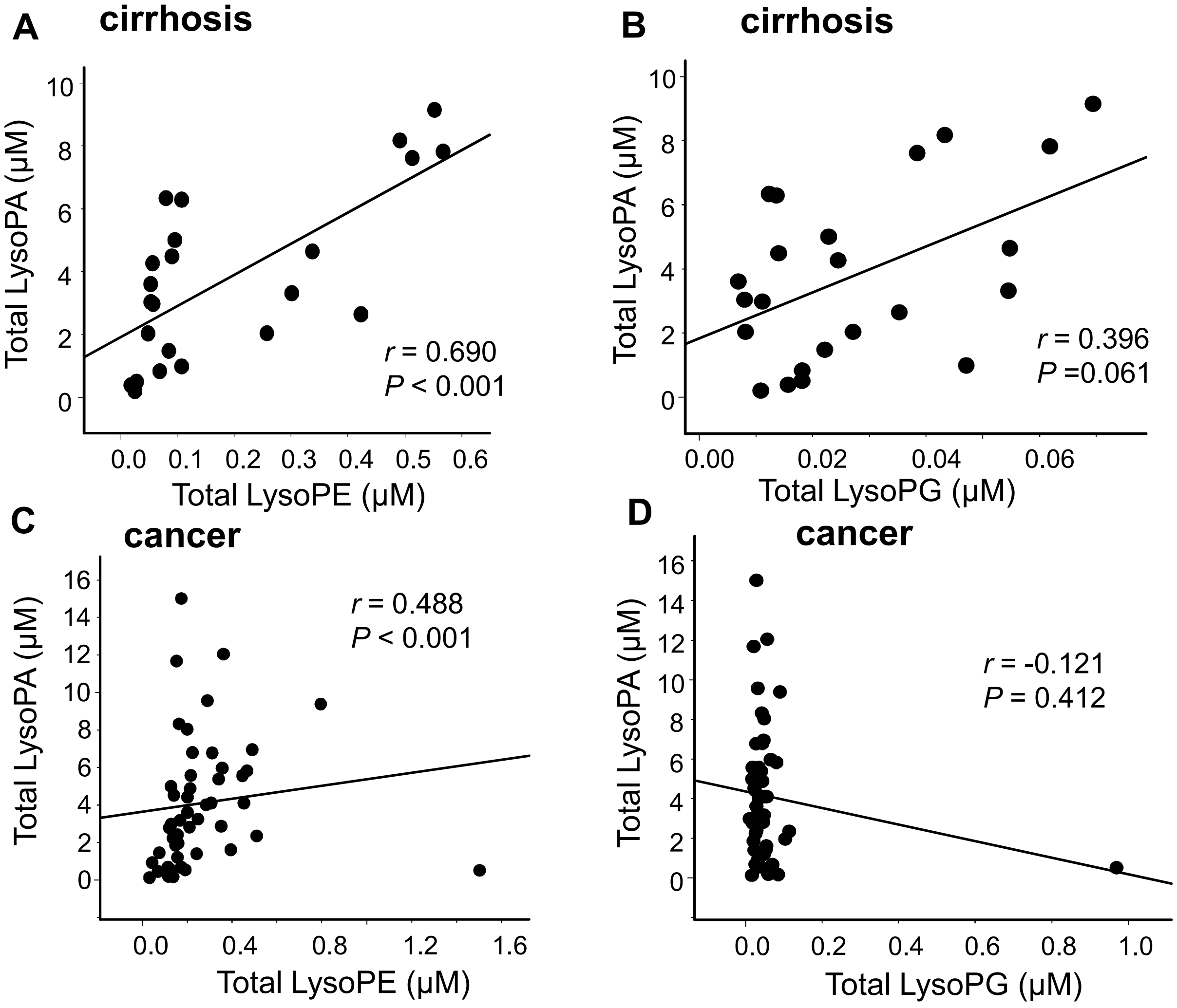

Supplemental Figure S4. Correlation between total LysoPA and total LysoPE or LysoPG levels in ascites from patients with cirrhosis or gastric cancer.
The correlations between the total LysoPA and total LysoPE (A, C) or total LysoPG (B, D) levels in ascites from patients with cirrhosis (A, B) or gastric cancer (C, D) are shown.

Supplemental Figure S5

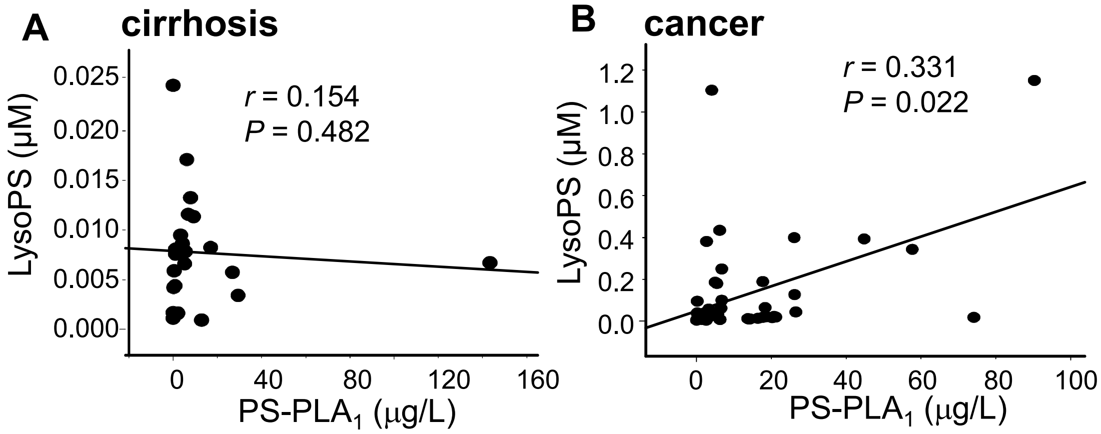

Supplemental Figure S5. Correlation between total LysoPS and PS-PLA1 levels in ascites from patients with cirrhosis or gastric cancer.
The correlations between the total LysoPS and PS-PLA1 levels were investigated in patients with cirrhosis (A) or gastric cancer (B).

Supplemental Table S1. Correlations between LysoPA molecular species and total ATX levels

	cirrhosis		cancer	
	r	***P***	***r***	***P***
16:0 LysoPA	0.809	<0.001	0.256	0.079
16:1 LysoPA	0.687	<0.001	0.376	0.008
18:0 LysoPA	0.796	<0.001	0.218	0.137
18:1 LysoPA	0.691	<0.001	0.345	0.016
18:2 LysoPA	0.668	<0.001	0.508	<0.001
20:4 LysoPA	0.726	<0.001	0.425	0.003
20:5 LysoPA	0.620	0.002	0.484	<0.001
22:5 LysoPA	0.724	<0.001	0.402	0.005
22:6 LysoPA	0.669	<0.001	0.467	0.001

The correlations between the LysoPA molecular species and the total ATX level were investigated using the Spearman correlation test.

Supplemental Table S2. Correlations between LysoPA molecular species and glycero-LPLs of the corresponding molecular species in ascites from patients with cirrhosis.

	LysoPE	LysoPG	LysoPI	LysoPC	LysoPS
16:0 LysoPA	0.671†	0.775†	0.890†	0.786†	
18:0 LysoPA	0.553†	0.679†	0.821†	0.189	0.300
18:1 LysoPA	0.479*	0.767†	0.835†	0.819†	0.519*
18:2 LysoPA	0.589†	0.542†	0.916†	0.869	
20:4 LysoPA	0.571†	-0.192	0.606†	0.189†	
22:6 LysoPA	0.809†	0.466*	0.700†	0.945†	

The correlations between the LysoPA molecular species and the glycero-LPLs of the corresponding molecular species were investigated using the Spearman correlation test. *$P < 0.05$, †$P < 0.01$.

Supplemental Figure S3. Correlations between LysoPA molecular species and glycero-LPLs of the corresponding molecular species in ascites from patients with gastric cancer.

	**LysoPE**	**LysoPG**	**LysoPI**	**LysoPC**	**LysoPS**
16:0 LysoPA	0.427†	0.192	0.437†	0.524†	
18:0 LysoPA	0.411†	0.145	0.550†	0.517	-0.281
18:1 LysoPA	0.235	-0.111	0.417†	0.587†	-0.562†
18:2 LysoPA	0.504†	-0.245	0.515†	0.705†	
20:4 LysoPA	0.571†	-0.192	0.606†	0.189	
22:6 LysoPA	0.733†	-0.062	0.581†	0.773†	

The correlations between the LysoPA molecular species and the glycero-LPLs of the corresponding molecular species were investigated using the Spearman correlation test. *$P<0.05$, †$P<0.01$
